# Comparative Clinical Performance of Multiple-Head Skin Prick Test Devices: A Prospective Evaluation of Pain, Sensitivity, Specificity, and Intradevice Variability

**DOI:** 10.7759/cureus.103646

**Published:** 2026-02-15

**Authors:** Marcos Sanchez-Gonzalez, Troy Grogan, Marvin Smollar, Syed A. A Rizvi

**Affiliations:** 1 Health Services Administration, Lake Erie College of Osteopathic Medicine, Bradenton, USA; 2 Research and Development, MedScience Research Group, West Palm Beach, USA; 3 Biomedical Sciences, Larkin University, Miami, USA

**Keywords:** allergy skin testing, device comparison, intradevice variability, multiple-head applicator, sensitivity, skin prick test, specificity

## Abstract

Background and objective

Multiple-head skin prick test (SPT) devices are essential tools for percutaneous allergy testing; however, significant interdevice variability exists that may affect diagnostic accuracy, clinical performance, and interpretation. Intradevice variability, characterized by inconsistent responses across individual test heads, may lead to false-positive or false-negative results, potentially compromising allergen identification and treatment planning. This prospective study compared four FDA-cleared multiple-head SPT devices with respect to pain perception, histamine sensitivity, glycerin specificity, and intradevice variability to identify clinically meaningful performance differences.

Methods

Thirty healthy adults participated in this institutional review board-approved study (approval 33-098). Participants were tested with two 10-head devices (AllerTest-10 (MedScience Research Group, Inc., West Palm Beach, FL, USA; distributed by ALK-Abelló, Inc., Port Washington, NY, USA) and Skintestor OMNI (Greer Laboratories, Inc., Lenoir, NC, USA)) and two eight-head devices (AllerTest-8 (MedScience Research Group, Inc.; distributed by ALK-Abelló, Inc.) and Multi-Test II (Lincoln Diagnostics, Inc., Decatur, IL, USA)). Pain was assessed using a Visual Analog Scale (VAS; 0-10). Histamine (1 mg/mL) and glycerin controls were applied to volar forearm surfaces. Sensitivity was defined as a histamine wheal ≥3 mm, and specificity as a glycerin wheal <3 mm. Intradevice variability was expressed as the coefficient of variation (CV) across test heads.

Results

Pain scores were low across all devices (mean VAS, 0.92-1.77), with no statistically significant differences. Among the 10-head devices, AllerTest-10 demonstrated superior sensitivity (88% vs. 68%; P = 0.002) and significantly lower intradevice variability (median CV, 0.18 vs. 0.72; P = 0.00024) compared with Skintestor OMNI. Among the eight-head devices, AllerTest-8 showed higher sensitivity (96% vs. 88%; P = 0.026) than Multi-Test II. Both AllerTest devices achieved 100% specificity, whereas comparators achieved 97%-98%. AllerTest devices demonstrated consistently low CV values (0.18-0.19), indicating superior test-head uniformity.

Conclusions

All evaluated devices demonstrated acceptable patient comfort and clinical performance. However, AllerTest devices exhibited superior clinical performance, characterized by higher histamine sensitivity, perfect glycerin specificity, and markedly lower intradevice variability. These findings suggest that device selection may meaningfully affect diagnostic accuracy, with lower intradevice variability reducing the likelihood of physician misinterpretation and improving the reliability of allergen identification.

## Introduction

Allergic diseases represent a substantial public health burden, affecting approximately two in five Americans and accounting for over 17 million physician visits, 30,000 emergency department visits, and hundreds of deaths annually [1-3]. The clinical and economic impact extends beyond direct healthcare utilization to include pharmaceutical costs, lost productivity, and diminished quality of life [4,5]. Given that allergic reactions are typically acquired, predictable, and rapid in onset, accurate identification of triggering allergens remains the cornerstone of effective diagnosis and treatment planning [6-10].

The skin prick test (SPT) has served as the primary diagnostic modality for immediate IgE-mediated allergic diseases since Charles Blackley first demonstrated its utility in the 19th century [11,12]. Contemporary practice has evolved toward multiple-head SPT devices that enable simultaneous testing of multiple allergens, potentially reducing patient discomfort and testing time [11]. These devices allow clinicians to efficiently develop allergen-avoidance strategies and prescribe allergen-specific immunotherapy when appropriate [1]. However, substantial performance variability exists among commercially available SPT devices, with important implications for clinical performance and diagnostic accuracy [13-16].

Intradevice variability, characterized by inconsistent responses across individual test heads within a single device, represents a particularly concerning phenomenon that may lead to physician misinterpretation and inappropriate treatment decisions. Intradevice variability can arise from manufacturing factors, including tooling mold precision, molding equipment quality, and production process controls [15,16]. Prior research from our group demonstrated that devices with lower finished-product variability, as assessed by high-magnification dimensional analysis, exhibited improved clinical performance, including reduced false-positive reactions to negative controls [17].

Accordingly, the present study extends this work by comparing four FDA-cleared multiple-head SPT devices across multiple clinically relevant endpoints, including patient-reported pain, histamine sensitivity, glycerin specificity, and intradevice variability. We hypothesized that devices demonstrating lower intradevice variability would exhibit superior clinical performance, thereby reducing the likelihood of diagnostic misinterpretation.

Specifically, this study was designed to evaluate whether measurable differences in device manufacturing precision translate into clinically meaningful performance outcomes, including pain perception, histamine sensitivity, glycerin specificity, and intradevice variability. By directly linking structural manufacturing characteristics to functional clinical metrics, we aimed to determine whether device precision materially influences diagnostic reliability in SPT.

## Materials and methods

Study design and ethics approval

This study was conducted in accordance with the ethical principles outlined in the Belmont Report, which serves as the foundational framework identified in the institution’s Federalwide Assurance for the Protection of Human Subjects, as well as the principles of the Declaration of Helsinki and applicable federal regulations governing human subjects research (45 CFR 46). Institutional review board approval was obtained prior to study initiation (approval 33-098). All participants provided written informed consent before enrollment. The study was prospectively registered at ClinicalTrials.gov (identifier: NCT07400718) prior to participant recruitment. The registry entry includes detailed information regarding study design, objectives, endpoints, and methodology to ensure transparency, reproducibility, and public accountability. All procedures were performed in accordance with the approved protocol, and no protocol deviations affecting participant safety, rights, or data integrity occurred during the study.

Participants

A total of 30 healthy adults aged 18-65 years were enrolled. Inclusion criteria were adults aged 18-65 years affiliated with the Faculty of Medicine, without known prior allergic conditions, without chronic skin conditions affecting the forearms, and able to provide informed consent. Participants were allocated to either the 10-head device comparison (n = 13) or the eight-head device comparison (n = 17). Exclusion criteria included a history of anaphylactic shock, acute febrile illness, chronic systemic disease manifestations, pregnancy, chronic skin conditions affecting the forearms, and antihistamine use within seven days of testing.

Test devices

Four FDA-cleared multiple-head SPT devices were evaluated in two paired comparisons. The 10-head comparison included AllerTest-10 (MedScience Research Group, Inc., West Palm Beach, FL, USA; distributed by ALK-Abelló, Inc., Port Washington, NY, USA) and Skintestor OMNI (Greer Laboratories, Inc., Lenoir, NC, USA). The eight-head comparison included AllerTest-8 (MedScience Research Group, Inc.; distributed by ALK-Abelló, Inc.) and Multi-Test II (Lincoln Diagnostics, Inc., Decatur, IL, USA).

Testing procedure

Histamine dihydrochloride (1 mg/mL; ALK-Abelló, Inc.) served as the positive control, and glycerin solution (ALK-Abelló) served as the negative control. Applications were performed on the volar surfaces of both forearms using new, sterile applicators for each test site. Test sites were maintained at least 2 cm apart to prevent cross-contamination between histamine and glycerin reactions.

One technician performed all device applications, and two independent technicians measured all wheal reactions. For analysis, the mean of the two readers’ measurements was used. If the two readings differed by ≥2 mm, both technicians reread the site, and a revised set of measurements was recorded. Testing technicians were blinded to solution contents and device assignments. A separate technician, who was not present during application, recorded all measurements.

All devices were applied using a standardized technique. Gentle, perpendicular pressure was applied until the device fully contacted the skin surface and resistance was met (i.e., until the device bottomed out), ensuring consistent penetration depth across applications. No rotational or rocking motion was used. Application force was standardized across participants and devices to minimize operator-dependent variability.

Outcome measures

Pain was assessed immediately following device application using a Visual Analog Scale (VAS) ranging from 0 (no pain) to 10 (unbearable pain). A clinically meaningful pain threshold was defined as a VAS score >4, indicating more than mild pain. Wheal measurements were obtained 15-20 minutes after application and recorded as mean diameter in millimeters.

Clinical performance was assessed using standard thresholds: sensitivity was calculated as the proportion of histamine sites producing wheals ≥3 mm, and specificity as the proportion of glycerin sites producing wheals <3 mm. Intradevice variability was quantified using the coefficient of variation (CV) calculated across all test heads for histamine wheal responses within each subject, with lower values indicating greater consistency.

Statistical analysis

Descriptive statistics are presented as mean ± SD or median with IQR, as appropriate. Pain scores were compared using the Wilcoxon signed-rank test for paired comparisons. Sensitivity and specificity proportions were compared using McNemar’s test. Intradevice CV values were compared using the Mann-Whitney U test. All analyses were conducted using IBM SPSS Statistics for Windows, Version 26.0 (Released 2018; IBM Corp., Armonk, NY, USA). A two-sided P-value <0.05 was considered statistically significant. Confidence intervals for proportions were calculated using the exact binomial (Clopper-Pearson) method.

## Results

Pain outcomes

**Figure 1:**
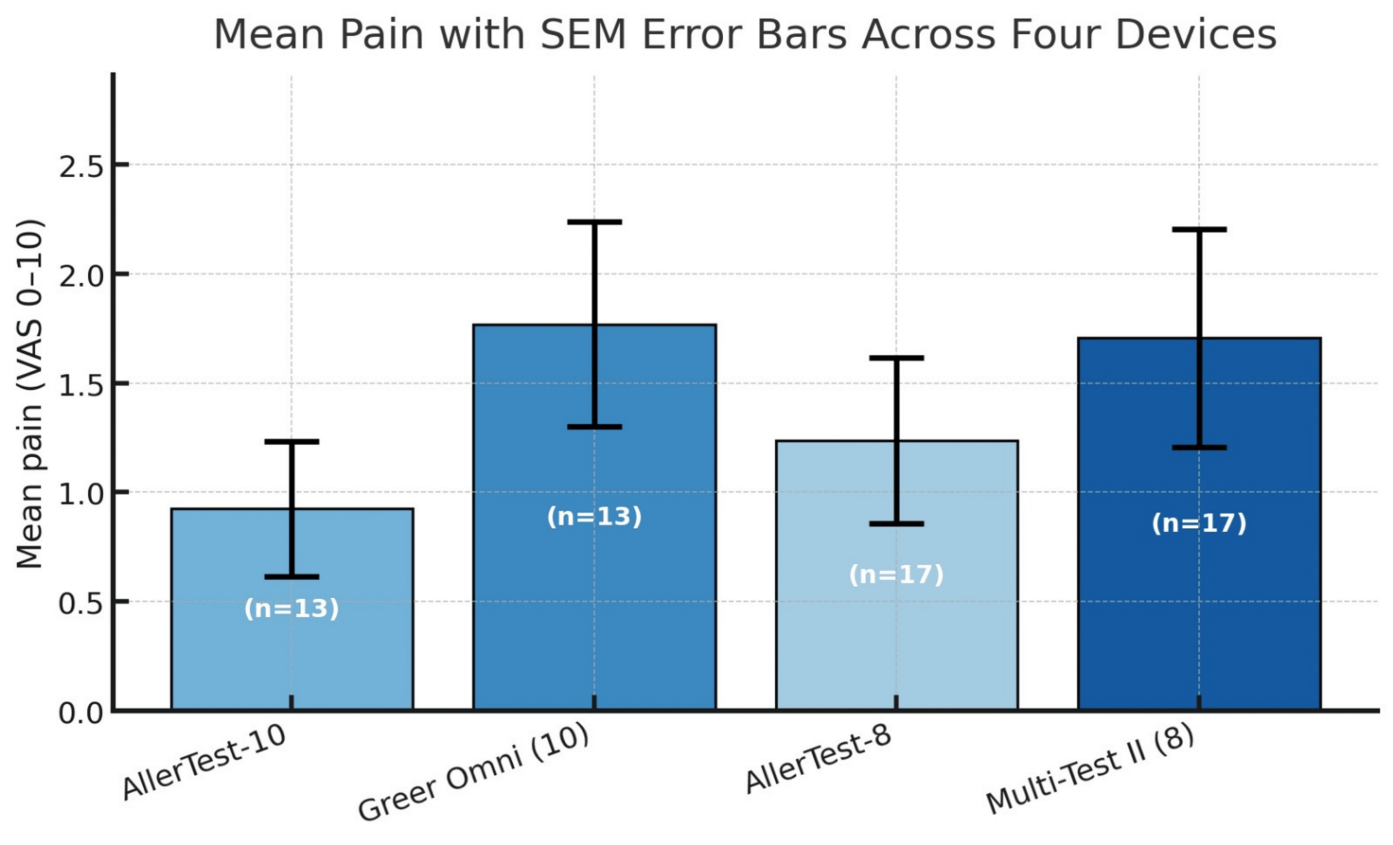
Mean pain scores (VAS 0-10) with SEM across four SPT devices AllerTest devices (AllerTest-10 and AllerTest-8) showed numerically lower pain scores than their respective comparators (Skintestor OMNI and Multi-Test II), though the differences did not reach statistical significance. SPT, skin prick test; VAS, Visual Analog Scale

Pain scores were uniformly low across all four devices, with mean VAS values ranging from 0.92 to 1.77 (Table 1, Figure 1). In the 10-head comparison, AllerTest-10 demonstrated a numerically lower mean pain score (0.92) than Skintestor OMNI (1.77), though this difference did not reach statistical significance (P = 0.085). No participants exceeded the VAS >4 threshold with AllerTest-10 (0%; 95% CI, 0-25%), whereas one participant did with Skintestor OMNI (8%; 95% CI, 0-36%).

**Table 1:** Pain outcome measures across devices (VAS 0-10) Pain >4 represents the proportion of subjects exceeding the “more than mild pain” threshold. VAS, Visual Analog Scale

Device	Mean pain (SD)	Median pain	Pain >4 (95% CI)
AllerTest-10 (n = 13)	0.92	1	0% (0-25)
Skintestor OMNI (n = 13)	1.77	2	8% (0-36)
AllerTest-8 (n = 17)	1.24	1	6% (0-29)
Multi-Test II (n = 17)	1.71	1	18% (4-43)

For the eight-head comparison, mean pain scores were 1.24 for AllerTest-8 and 1.71 for Multi-Test II (P = 0.066). Both devices had median pain scores of 1. One participant (6%; 95% CI, 0-29%) exceeded the VAS >4 threshold with AllerTest-8, compared to three participants (18%; 95% CI, 4-43%) with Multi-Test II. Overall, all devices demonstrated pain profiles consistent with well-tolerated testing procedures.

Clinical performance: sensitivity and specificity

**Figure 2:**
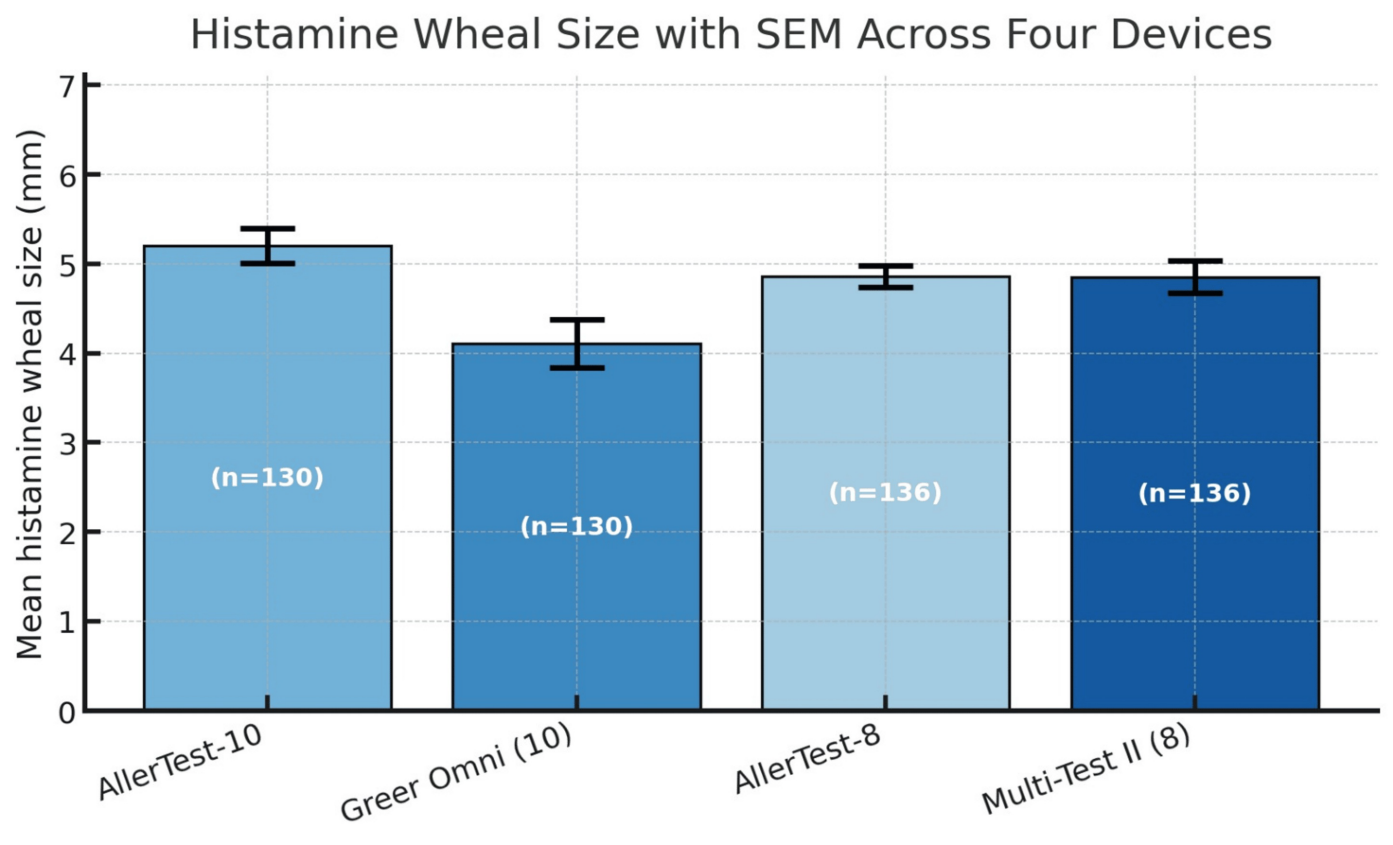
Mean histamine wheal size (mm) with SEM across four SPT devices AllerTest-10 produced the largest mean wheal size (5.2 mm), while Skintestor OMNI demonstrated the lowest (4.1 mm) with greater variability. All devices produced positive control responses exceeding the 3 mm threshold for clinical significance. SPT, skin prick test

**Figure 3:**
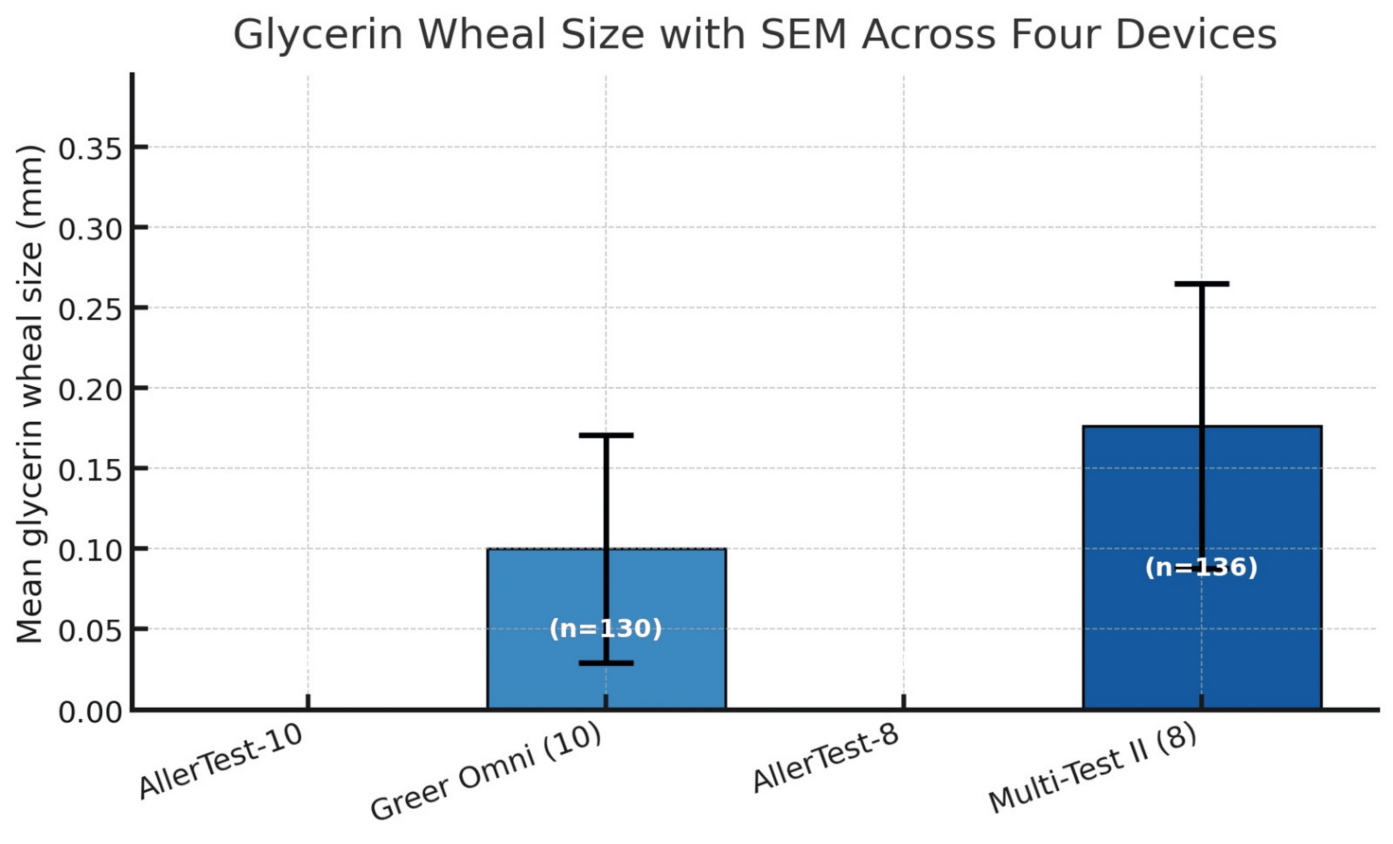
Mean glycerin (negative control) wheal size (mm) with SEM across four SPT devices Both AllerTest devices demonstrated zero glycerin wheal responses (perfect negative control performance), whereas Skintestor OMNI and Multi-Test II showed small nonzero means driven by occasional outlying sites. SPT, skin prick test

Clinical performance metrics demonstrated meaningful differences between devices (Table 2, Figure 2). In the 10-head comparison (130 test sites per device), AllerTest-10 achieved a sensitivity of 88% (95% CI, 82-93%) compared to 68% (95% CI, 60-76%) for Skintestor OMNI (P = 0.002). Specificity was 100% (95% CI, 97-100%) for AllerTest-10 and 98% (95% CI, 95-100%) for Skintestor OMNI. Mean histamine wheal size was larger for AllerTest-10 (5.2 ± 2.2 mm) than Skintestor OMNI (4.1 ± 3.1 mm), while glycerin responses were essentially absent for AllerTest-10 (0.0 ± 0.0 mm) versus minimal for Skintestor OMNI (0.1 ± 0.8 mm) (Figure 3).

**Table 2:** Clinical performance for multiple-head SPT devices Values are presented as mean ± SD for wheal sizes. Sensitivity = proportion of histamine sites with wheal ≥3 mm. Specificity = proportion of glycerin sites with wheal <3 mm. 95% CIs are exact binomial (Clopper-Pearson). 10-head devices: n = 130 sites per device; eight-head devices: n = 136 sites per device.

Device	Histamine (mm)	Glycerin (mm)	Sensitivity % (95% CI)	Specificity % (95% CI)
AllerTest-10	5.2 ± 2.2	0.0 ± 0.0	88% (82-93)	100% (97-100)
Skintestor OMNI	4.1 ± 3.1	0.1 ± 0.8	68% (60-76)	98% (95-100)
AllerTest-8	4.86 ± 1.44	0.00 ± 0.00	96% (91-98)	100% (97-100)
Multi-Test II	4.85 ± 2.14	0.18 ± 1.03	88% (81-92)	97% (93-99)

In the eight-head comparison (136 test sites per device), AllerTest-8 demonstrated a sensitivity of 96% (95% CI, 91-98%) compared to 88% (95% CI, 81-92%) for Multi-Test II (P = 0.026). Specificity was 100% (95% CI, 97-100%) for AllerTest-8 and 97% (95% CI, 93-99%) for Multi-Test II. Mean histamine wheal sizes were comparable between AllerTest-8 (4.86 ± 1.44 mm) and Multi-Test II (4.85 ± 2.14 mm), though AllerTest-8 demonstrated narrower variability. Glycerin responses were absent for AllerTest-8 (0.00 ± 0.00 mm) and minimal for Multi-Test II (0.18 ± 1.03 mm) (Figure 3).

Intradevice variability

CV analysis revealed substantial differences in intradevice consistency (Table 3). AllerTest-10 demonstrated the lowest variability with a median CV of 0.18 (IQR, 0.08-0.40), significantly lower than Skintestor OMNI at 0.72 (IQR, 0.57-0.75; P = 0.00024), representing a four-fold difference in intradevice consistency. AllerTest-8 showed similarly low variability (median CV, 0.19; IQR, 0.16-0.26), comparable to Multi-Test II (median CV, 0.21; IQR, 0.18-0.65; P = 0.179).

**Table 3:** Intradevice variability expressed as CV CV was calculated across test heads for histamine wheal size; lower values indicate greater intradevice consistency. CV, coefficient of variation

Device	Histamine wheal (mm)	CV Median (IQR)
AllerTest-10	5.2 ± 2.2	0.18 (0.08-0.40)
Skintestor OMNI	4.1 ± 3.1	0.72 (0.57-0.75)
AllerTest-8	4.9 ± 1.4	0.19 (0.16-0.26)
Multi-Test II	4.9 ± 2.1	0.21 (0.18-0.65)

Statistical comparison summary

Table 4 summarizes the paired statistical comparisons across all outcome measures. No statistically significant differences in pain perception were observed between paired devices, indicating comparable patient tolerability. In contrast, significant differences were identified in histamine sensitivity for both device comparisons, with AllerTest-10 demonstrating higher sensitivity than Skintestor OMNI (P = 0.002) and AllerTest-8 outperforming Multi-Test II (P = 0.026).

**Table 4:** Summary of paired device comparison P-values ^*^ Statistically significant (P < 0.05) CV, coefficient of variation

Comparison	Pain P	Sensitivity P	Specificity P	CV P
AllerTest-10 vs. Skintestor OMNI	0.085	0.002^*^	0.317	0.00024*
AllerTest-8 vs. Multi-Test II	0.066	0.026^*^	0.180	0.179

A marked difference in intradevice variability was observed in the 10-head comparison, where AllerTest-10 exhibited a significantly lower CV than Skintestor OMNI (P = 0.00024), reflecting greater test-head consistency. No significant differences in specificity were observed between paired devices, and intradevice variability did not differ significantly in the eight-head comparison. Overall, these findings indicate that performance differences among devices were driven primarily by sensitivity and intradevice consistency rather than by patient comfort. 

## Discussion

The present study evaluated four FDA-cleared multiple-head SPT devices in two paired comparisons across clinically relevant endpoints, including patient-reported pain, histamine sensitivity, glycerin specificity, and intradevice variability. Results of this head-to-head evaluation demonstrate measurable performance differences with potential implications for diagnostic reliability in allergy testing. Although all devices demonstrated favorable tolerability with low pain scores, marked differences were observed in histamine sensitivity and intradevice variability, parameters that directly influence test interpretation and the consistency of clinical decision-making.

The most striking finding was the pronounced difference in intradevice variability between AllerTest-10 and Skintestor OMNI, with AllerTest-10 demonstrating a fourfold lower CV (0.18 vs. 0.72; P = 0.00024). Such variability suggests that individual test heads may deliver inconsistent allergen penetration depth, potentially leading to discordant wheal responses across heads within the same device. From a diagnostic standpoint, this inconsistency may introduce uncertainty in result interpretation and could necessitate repeat testing under certain circumstances.

These findings align with and extend our previous work demonstrating that manufacturing precision, specifically tine length and diameter consistency, correlates with clinical performance [17]. The Skintestor OMNI’s higher variability and lower sensitivity (68% vs. 88%) likely reflect greater dimensional inconsistency among test heads, a hypothesis supported by the device’s larger SD in histamine wheal sizes (3.1 mm vs. 2.2 mm). In contrast, both AllerTest devices demonstrated narrow variability profiles consistent with tighter manufacturing tolerances.

Although prior reports have shown variable performance across SPT devices, our findings indicate that, under the standardized conditions of the present study, differences in sensitivity and control reactivity may have clinically meaningful implications for diagnostic accuracy. Importantly, this study was not designed to directly measure downstream clinical outcomes. However, the observed improvements in intradevice consistency and control performance suggest potential benefits in reducing false-negative or false-positive test interpretations in clinical practice. The lower histamine sensitivity observed with Skintestor OMNI suggests a greater potential for false-negative responses under these testing conditions [11,13,16,17], which could contribute to missed allergen identification. Conversely, the absence of glycerin reactions observed with the AllerTest devices in this cohort suggests high control specificity, potentially reducing the likelihood of false-positive interpretations and unnecessary downstream interventions related to nonspecific reactivity.

Prior studies comparing SPT devices have reported variable results, though differences in methodology, patient populations, and devices evaluated complicate direct comparisons. Carr et al. (2005) reported Multi-Test II sensitivity of 93% and specificity of 99% [14], somewhat higher than our findings of 88% and 97%, respectively. This modest discrepancy may reflect differences between tightly controlled research environments and real-world testing conditions in our study. Dykewicz et al. (2007) compared Multi-Test II and Skintestor OMNI, reporting sensitivities of 93% and 94%, respectively [18], which exceed those observed in our Skintestor OMNI evaluation. These differences underscore the importance of independent, head-to-head evaluations under standardized protocols. Collectively, these data reinforce the observation that device-specific engineering characteristics may influence test reliability across clinical settings [13-15,17,18].

Pain perception was uniformly low across all devices, with no statistically significant differences in paired comparisons. This finding is consistent with prior reports indicating that SPT is generally well tolerated regardless of device selection [14,17]. The absence of meaningful differences in patient-reported discomfort suggests that device selection may be more appropriately guided by reproducibility and diagnostic performance metrics, including sensitivity, specificity, and intradevice consistency, rather than by tolerability alone.

Several limitations warrant consideration. The sample size, while adequate to detect the observed differences in sensitivity and variability, may have been underpowered to identify smaller differences in pain perception or specificity. Testing was conducted at a single site and within a limited temporal window, which may not fully capture operator-dependent or day-to-day variability. Additionally, evaluation was limited to histamine and glycerin controls; performance characteristics with allergen extracts of varying potency and stability were not assessed. Finally, one subject in the 10-head comparison demonstrated near-absent histamine reactivity across both devices, consistent with the rare phenomenon of histamine nonresponse; sensitivity analysis excluding this subject yielded even higher performance for AllerTest-10 (94% sensitivity).

Collectively, these findings underscore the importance of device-level precision in allergy diagnostics and suggest that manufacturing consistency may represent an underrecognized determinant of test reliability. Further studies in allergic populations and in real-world clinical workflows are warranted to determine how these device-level performance characteristics translate into broader diagnostic practice.

## Conclusions

This study demonstrated performance differences among commercially available multihead SPT devices evaluated under standardized conditions. While all devices exhibited broadly acceptable function and tolerability, measurable differences were observed in sensitivity, specificity, and intradevice consistency. Where such differences emerged, they may reflect underlying engineering characteristics, particularly tine sharpness, tine diameter, and the resulting consistency of skin penetration, as suggested by prior studies. Within this context, AllerTest devices in both eight-head and 10-head configurations demonstrated higher histamine sensitivity, absence of glycerin reactivity in this cohort, and lower intradevice variability compared with their respective comparators. The approximately fourfold lower CV observed with AllerTest-10 relative to Skintestor OMNI is clinically relevant, as reduced intradevice variability may decrease interpretive ambiguity and support more consistent allergen identification.

Importantly, this study was not designed to directly assess downstream clinical outcomes. However, improved reproducibility and control performance characteristics may enhance diagnostic confidence and reduce false-positive or false-negative interpretations in routine clinical practice. Device selection for percutaneous allergy testing should therefore consider not only regulatory clearance status but also demonstrated performance characteristics, including sensitivity, specificity, and intradevice variability. Devices exhibiting lower variability and stable control reactivity may offer advantages in diagnostic reliability, particularly in high-volume or community practice settings. Future studies examining device performance with actual allergen extracts in allergic populations, as well as multicenter evaluations incorporating operator variability, will further clarify how device-level engineering characteristics influence real-world diagnostic performance.

## References

[1] Cox L, Williams B, Sicherer S (2008). Pearls and pitfalls of allergy diagnostic testing: report from the American College of Allergy, Asthma and Immunology/American Academy of Allergy, Asthma and Immunology Specific IgE Test Task Force. Ann Allergy Asthma Immunol.

[2] Bock SA, Muñoz-Furlong A, Sampson HA (2007). Further fatalities caused by anaphylactic reactions to food, 2001-2006. J Allergy Clin Immunol.

[3] Seité S, Kuo AM, Taieb C, Strugar TL, Lio P (2020). Self-reported prevalence of allergies in the USA and impact on skin—an epidemiological study on a representative sample of American adults. Int J Environ Res Public Health.

[4] Fritzsching B, Contoli M, Porsbjerg C (2022). Long-term real-world effectiveness of allergy immunotherapy in patients with allergic rhinitis and asthma: results from the REACT study, a retrospective cohort study. Lancet Reg Health Eur.

[5] Fong AT, Ahlstedt S, Golding MA, Protudjer JL (2022). The economic burden of food allergy: what we know and what we need to learn. Curr Treat Options Allergy.

[6] Mäntylä J, Thomander T, Hakulinen A (2018). The effect of oral immunotherapy treatment in severe IgE mediated milk, peanut, and egg allergy in adults. Immun Inflamm Dis.

[7] LaHood NA, Patil SU (2019). Food allergy testing. Clin Lab Med.

[8] Peters RL, Allen KJ, Dharmage SC (2013). Skin prick test responses and allergen-specific IgE levels as predictors of peanut, egg, and sesame allergy in infants. J Allergy Clin Immunol.

[9] Lomidze N, Gotua T, Gotua M (2015). Ige-mediated food allergy - current problems and future perspectives (review). Georgian Med News.

[10] Kianifar HR, Pourreza A, Jabbari Azad F, Yousefzadeh H, Masomi F (2016). Sensitivity comparison of the skin prick test and serum and fecal radio allergosorbent test (RAST) in diagnosis of food allergy in children. Rep Biochem Mol Biol.

[11] Bernstein IL, Li JT, Bernstein DI (2008). Allergy diagnostic testing: an updated practice parameter. Ann Allergy Asthma Immunol.

[12] (2016). Skin testing for allergic rhinitis: a health technology assessment. Ont Health Technol Assess Ser.

[13] Matsui EC, Keet CA (2015). Are all skin testing devices created equal?. J Allergy Clin Immunol Pract.

[14] Carr WW, Martin B, Howard RS, Cox L, Borish L (2005). Comparison of test devices for skin prick testing. J Allergy Clin Immunol.

[15] Werther RL, Choo S, Lee KJ, Poole D, Allen KJ, Tang ML (2012). Variability in skin prick test results performed by multiple operators depends on the device used. World Allergy Organ J.

[16] Heinzerling L, Mari A, Bergmann KC (2013). The skin prick test - European standards. Clin Transl Allergy.

[17] Sanchez-Gonzalez MA, Grogan TJ, Smollar M, Rizvi SA (2023). Improving allergy testing and diagnosis: impact of skin prick testing intra-head device variability on clinical performance. J Prevent Complement Med.

[18] Dykewicz MS, Lemmon JK, Keaney DL (2007). Comparison of the Multi-Test II and Skintestor Omni allergy skin test devices. Ann Allergy Asthma Immunol.

